# O7-7 School-based intervention and nutritional behaviours change among adolescents: cross-sectional latent class and longitudinal latent transition analysis

**DOI:** 10.1093/eurpub/ckac094.055

**Published:** 2022-08-29

**Authors:** Mohamed Dakin, Florian Manneville, Abdou Omorou

**Affiliations:** 1 APEMAC, University of Lorraine, Vandoeuvre-Lès-Nancy, France; 2 CHRU de Nancy, Vandoeuvre-Lès-Nancy, France; 3 Nancy Publique Health School, University of Lorraine, Vandoeuvre-Lès-Nancy, France

**Keywords:** Diet, Physical activity, Sedentary behaviour, Adolescent, latent class, latent transition

## Abstract

**Background:**

Nutritional behaviours such as diet, physical activity (PA) and sedentary behaviour (SB) are interdependent and are likely to change in different way during a public health intervention. This study aimed to 1) identify cross-sectional nutritional profiles and their 2-year longitudinal transition among French school-aged adolescents, 2) identify factors associated with these profiles and transition.

**Methods:**

Adolescents from the 2-year school-based PRALIMAP (PRomotion de l'ALIMentation et de l'Activité Physique) intervention were included. Nutritional behaviours (diet, PA and SB) were assessed by self-administered questionnaire at the beginning (T0) and end (T2) of the study. Nutritional profiles were identified at T0 using latent class analysis, and their transition from T0 to T2 using latent transition analysis. Logistic regression models were computed to identify associated factors such as sex, weight status, age, socioeconomic status and a PRALIMAP intervention.

**Results:**

Among the 2390 adolescents included (mean ± *SD* age=15.1 ± 0.6 years), 5 nutritional profiles were evidenced at T0 and labelled as ‘healthy'(12.2%), ‘excessive diet' (16.0%), ‘physically inactive' (26.1%), ‘restrictive diet' (23.5%), and ‘unhealthy' (22.2%). Compared to ‘physically inactive' profile, adolescents from the ‘healthy' ones were less likely to be girls (OR = 0.21; p>.0001), and socially advantaged (OR = 0.87; p=.0002). ‘Unhealthy' profile adolescents were less likely to be overweight/obese (OR = 0.48; p>.0001) and more likely to be older (OR = 3.67; p>.0001). At T2, proportion of adolescents increased in ‘healthy' (+4.6%), ‘unhealthy' (+2.8%) profiles, and decreased in ‘excessive diet' (-1.2%), ‘physically inactive' (-3.2%), ‘restrictive diet' (-3.03%) profiles. Compared to the control group, adolescents from the intervention group were more likely to remain in ‘healthy' (OR = 1.3), and to switch from ‘excessive eater' (OR = 1.1) and ‘physically inactive' (OR = 2.6) to ‘healthy'. Sustainability of ‘healthy' profile increased with socioeconomic status.

**Conclusions:**

The results of this study confirm the variety of behavioural profiles in nutrition with significant social differences. It also demonstrated the effectiveness of the intervention in changing behaviours favourably. Public health focusing on nutritional changes may be more effective it taking in to account the social status of adolescents.

